# CloudATAC: a cloud-based framework for ATAC-Seq data analysis

**DOI:** 10.1093/bib/bbae090

**Published:** 2024-07-23

**Authors:** Avinash M Veerappa, M Jordan Rowley, Angela Maggio, Laura Beaudry, Dale Hawkins, Allen Kim, Sahil Sethi, Paul L Sorgen, Chittibabu Guda

**Affiliations:** University of Nebraska Medical Center, Omaha, NE 68105 USA; University of Nebraska Medical Center, Omaha, NE 68105 USA; Deloitte Consulting LLP, Health Data and AI Arlington, VA, USA; Google Google Public Sector, Professional Services Reston, VA, USA; Google Google Public Sector, Professional Services Reston, VA, USA; Google Google Public Sector, Professional Services Reston, VA, USA; University of Nebraska Medical Center, Omaha, NE 68105 USA; University of Nebraska Medical Center, Omaha, NE 68105 USA; University of Nebraska Medical Center, Omaha, NE 68105 USA

**Keywords:** ATACseq, single-cell ATAC, chromatin, Google Cloud, NIH strides, NIH Cloud Lab

## Abstract

Assay for transposase-accessible chromatin with high-throughput sequencing (ATAC-seq) generates genome-wide chromatin accessibility profiles, providing valuable insights into epigenetic gene regulation at both pooled-cell and single-cell population levels. Comprehensive analysis of ATAC-seq data involves the use of various interdependent programs. Learning the correct sequence of steps needed to process the data can represent a major hurdle. Selecting appropriate parameters at each stage, including pre-analysis, core analysis, and advanced downstream analysis, is important to ensure accurate analysis and interpretation of ATAC-seq data. Additionally, obtaining and working within a limited computational environment presents a significant challenge to non-bioinformatic researchers. Therefore, we present Cloud ATAC, an open-source, cloud-based interactive framework with a scalable, flexible, and streamlined analysis framework based on the best practices approach for pooled-cell and single-cell ATAC-seq data. These frameworks use on-demand computational power and memory, scalability, and a secure and compliant environment provided by the Google Cloud. Additionally, we leverage Jupyter Notebook's interactive computing platform that combines live code, tutorials, narrative text, flashcards, quizzes, and custom visualizations to enhance learning and analysis. Further, leveraging GPU instances has significantly improved the run-time of the single-cell framework. The source codes and data are publicly available through NIH Cloud lab https://github.com/NIGMS/ATAC-Seq-and-Single-Cell-ATAC-Seq-Analysis.

This manuscript describes the development of a resource module that is part of a learning platform named ``NIGMS Sandbox for Cloud-based Learning'' https://github.com/NIGMS/NIGMS-Sandbox. The overall genesis of the Sandbox is described in the editorial NIGMS Sandbox [1] at the beginning of this Supplement. This module delivers learning materials on the analysis of bulk and single-cell ATAC-seq data in an interactive format that uses appropriate cloud resources for data access and analyses.

## INTRODUCTION

ATAC-seq, short for Assay for Transposase-Accessible Chromatin with high-throughput sequencing [2], uses hyperactive Tn5 transposases to insert adapters into regions of accessible chromatin enabling high-throughput sequencing of the intervening DNA. Sequenced reads from ATAC-seq experiments are used to detect regions of accessible chromatin, such as at active promoters or enhancers. ATAC-seq data can also be used to identify transcription factor binding sites (TFBS) through footprinting and to measure the positioning of nucleosomes. The ATAC-seq method was developed by Buenrostro and Giresi [3] and since then has evolved to include sequencing at single-cell resolution (scATAC-seq) [4, 5]. Several technological variations have emerged for ATAC-seq, such as SnapATAC and Droplet-based scATAC-seq. The bioinformatic analyses of any variant of ATAC-seq often involve several core components, including quality check, preprocessing, alignment, peak calling, differential peak identification, annotation, motif enrichment, footprinting and nucleosome position analysis. Furthermore, variations also exist at the level of bioinformatics workflows, with the development of tools such as epiConv [6], RAPIDS [7] and scATACpipe [8] to provide alternative approaches for analysis.

Performing ATAC-seq using conventional computational systems poses several challenges and limitations [9]. High-performance clusters are essential for processing large-scale genomic data, but maintaining and upgrading these systems can be expensive and laborious. Furthermore, the requirement for high-performance computing servers, terabytes to petabytes of storage, high capacity network bandwidth for data transfers, and reliance on IT personnel for systems support create added burden on the researchers and institutions. Finally, the bioinformatics expertise is crucial for working with appropriate tools, debugging computational frameworks, and optimizing workflows to extract meaningful insights from ATAC-seq data. The availability of such comprehensive expertise and infrastructure varies across research institutions, making it extremely challenging for those with limited resources to carry out such complex analyses, locally.

In contrast, utilizing cloud computing for ATAC-seq offers several advantages. Cloud platforms provide scalable and flexible computing resources on-demand, eliminating the need for perpetually maintaining expensive hardware [10–12]. Users can access and pay for the required computing power and storage capacity as needed, resulting in cost efficiency, and reduced financial constraints. Cloud services also offer high network bandwidth, enabling seamless data transfer and analysis, mainly when working with large genomic datasets. Therefore, leveraging cloud-based computational systems for ATAC-seq provides a scalable, cost-effective and streamlined approach, empowering users to accelerate their genomic studies and gain valuable insights into chromatin accessibility and epigenetic gene regulation.

Therefore, we introduce CloudATAC, a comprehensive module developed as part of the NIGMS Sandbox initiative. This module offers a streamlined, step-by-step walkthrough for efficiently analysing both pooled-cell and single-cell ATAC-seq data, effectively overcoming the aforementioned bottlenecks. Through specialized tools, live and customizable codes, best practices approaches, and embedded video segments, users will gain valuable and practical insights into the analysis process. Short quizzes are provided at the end of each section to reinforce understanding, allowing users to assess their knowledge and progress. The CloudATAC module is conveniently implemented in *Jupyter Notebooks*, ensuring an interactive and user-friendly experience and it is comprised of two independent submodules to work with the pooled- and single cell ATAC-seq datasets.

## METHODS

For the development of CloudATAC, we focused on utilizing Google Cloud, as decided by the NIH/NIGMS Sandbox initiative. This choice was driven by the need to maximize development speed and reduce complexity. Google Cloud's advantages in services like Vertex AI, Cloud Storage, and Cloud Run significantly contributed to this decision. We conducted a comprehensive evaluation of cloud platforms, finding Google Cloud's integration capabilities, computational scalability, workflow optimization tools, infrastructure management, and open-source support to be better. Table 1 shows the comparison among these factors across various cloud platforms. The CloudATAC module leverages various Google Cloud services, including Google Cloud Storage, Vertex AI workbench, *RAPIDS-AI*, Artifact Registry, Cloud Build and BigQuery. These cloud services were anchored with *JupyterLab* [13], *Docker* [14], *Git* [15] and software libraries such as *Bioconda Anaconda* [16] channel in *Python*.

**Table 1:** Comparison of features including integration, computational scalability, workflow optimization, infrastructure management, and open-source capabilities across various cloud platforms

Feature/Aspect	CloudATAC (Google Cloud)	Galaxy	Terra	Nextflow Tower
Integration and Environment	Integrated with Vertex AI for unified ML tasks. Jupyter Notebook for interactivity.	Requires additional steps or external tools for similar ML tasks. More complex to integrate interactivity.	Similar challenges in integration and environment flexibility.	Offers workflow management but may lack deep integration with specific ML and data processing tools.
Computational Scalability	High scalability with Cloud Storage and Cloud Run. GPU acceleration for faster processing.	Limited by the infrastructure it's deployed on. Less flexible in terms of scalability and GPU usage.	Variable scalability depending on the underlying cloud provider.	Scalable but depends on the underlying infrastructure and specific configurations.
Workflow Optimization	Preconfigured for ATAC-seq data. Offers optimized pipelines and GPU acceleration.	Flexible framework suitable for diverse workflows but requires building from scratch for optimization.	Designed for biomedical research but may need customization for ATAC-seq data.	Focused on batch processing and may require additional setup for optimized ATAC-seq analysis.
Learning and Visualization	Includes interactive modules, quizzes, and visualizations specific to ATAC-seq.	General-purpose visualization tools; may not be as tailored to ATAC-seq without customization.	Offers visualization tools but may require additional effort to tailor to ATAC-seq.	Provides visualization but may lack specific features for interactive learning in ATAC-seq.
Infrastructure Management	Streamlined management within Google Cloud ecosystem. Serverless services for genomics.	More complex configuration and infrastructure management required.	Requires management of cloud-based resources, which can be complex.	Simplifies workflow execution but may not offer as streamlined an infrastructure management.
Open Source and Portability	Docker containers and workflow code made open source for portability, despite initial Google Cloud-specific design.	Open platform for diverse workflows, with a strong emphasis on community and open-source tools.	Offers open-source tools but may have specific dependencies on cloud services.	Supports open-source workflows but may require adaptation for specific cloud environments.

### Google cloud storage

Google Cloud storage was used to create storage buckets to store example data files from the studies by Bao, Rubin [17] and Lareau, Duarte [18] for pooled-cell ATAC-seq and sc-ATAC-seq along with the reference and output files from the workflows. Controlled access to these files was employed through Identity and Access Management, while the Google Cloud console facilitated the browser-based visual interface for managing the data bucket.

### Augmenting ATAC-seq workflows utilizing the best practices approach

The pooled-cell (ATAC-seq) and single-cell (scATAC-seq) workflows were augmented by utilizing suitable programs with applicable parameters and providing flexibility for parameter tuning via the command line interface (CLI). The parameters are hard-coded into CLI that can be modified empirically for any context. The workflows, along with their visualization and user interface, have been designed with the needs of non-programmers in mind, enabling them to explore and analyze the data quickly. Tables 2 and 3 describe the tools with specific purposes and parameters to augment pooled-cell ATAC-seq and scATAC-seq analysis workflows, respectively. We used CPU-based architecture for augmenting pooled-cell ATAC-seq workflow, while a GPU-based architecture was employed for scATAC-seq to enable faster processing speeds with on-demand scalability to meet large matric calculations.

**Table 2:** An overview of various tools involved in processing and analyzing ATAC-seq data, including quality control, trimming, alignment, post-processing, peak identification, differential analysis, annotation, and visualization along with their parameters/functions used in developing the best practices approach for analyzing pooled-cell ATACseq data

Pipeline	Program/Tools	Purpose	Parameters/Function Used		Parameter Tuning
ATACseq	fastqc	Quality control tool for ATAC sequence data	-t, −q, −o,		Yes
	multiqc	Parsing results and statistics to summarize metrics	-f, −o		Yes
	Trimmomatic	Performs trimming of paired- and single-end data	PE, −threads, LEADING:, TRAILING:, MINLEN:		Yes
	Bowtie2	Aligns sequencing reads to reference sequences	-p, −x, −1, −2, -S		Yes
	samtools	Post-processing of alignments for converting the format to BAM and sorting	view, −q, −b, −h, −s, sort, −o, index		Yes
	Picard	Manipulating formats such as SAM/BAM/CRAM	*MarkDuplicates*	—REMOVE_DUPLICATES, -I, -O, —METRICS_FILE	Yes
	deepTools	Creating normalized coverage files in bigWig file formats	*bamCoverage*	-b, −o, −bs, 1 -p	Yes
	deepTools	Calculates scores per genome regions and prepares an intermediate file	*computeMatrix*	-S, −R, −a, −o, −m	Yes
	deepTools	Calculates the fragment sizes for read pairs	*bamPEFragmentSize*	-b, −o, −p, —maxFragmentLength	Yes
	deepTools	Shifting the reads	*alignmentSieve*	-p, −b, —ATACshift	Yes
	MACS2	Genome-wide peak identification	*callpeak*	-f, −g, —keep-dup, —cutoff-analysis, −n, −t	
	MAnorm	Quantitating peak signal differences	—p1, —p2, —r1, —r2, —rf, —n1, —n2, —pe, −w, −o, —wa		Yes
	HOMER	Annotation of differential peaks	*annotatePeaks.pl*	-annStats, −go	Yes
	TOBIAS	Correcting Tn5 insertion bias	*ATACorrect*	—bam —genome —peaks —outdir —prefix —cores —verbosity	Yes
	TOBIAS	Calculating footprint scores around peaks	*ScoreBigwig*	-s -r -o —cores —verbosity 1	Yes
	TOBIAS	Calculating statistics for motifs and differential footprint score	*BINDetect*	—signals —genome —peaks —outdir —cond_names —cores —verbosity 1	Yes
	TOBIAS	Visualizing the average footprint at TF motifs	*PlotAggregate*	—TFBS, —signals, —output, —share_y, —verbosity, —plot_boundaries, —flank, —smooth, —signal-on-x	Yes

**Table 3:** Overview of tools utilized in augmenting scATAC-seq workflow along with parameters and functions

Pipeline	Custom python files by NVIDIA	Custom python functions	Packages used	Purpose	Parameters Used	Parameter Tuning Enabled
scATACseq RAPIDS-AI	scATACseq_Tutorial4.ipynb	rmm	*rmm* *cuda*	Allowing for oversubscription of resources	managed_memory=, devices=	Yes
	scATACseq_Tutorial4.ipynb	Defining variables	-	Filtering peaks	n_top_peaks =	Yes
				PCA	n_components =	Yes
				t-SNE	tsne_n_pcs =	Yes
				KNN	n_neighbors =, knn_n_pcs =	Yes
				UMAP	umap_min_dist =, umap_spread =	Yes
				Differential peaks	n_diff_peaks =	Yes
	Marker genes	markers =	Yes			
	rapids_scanpy_funcs.py	read_with_filter	-	Reads an h5ad file and applies cell and genes count filter	min_genes_per_cell=, max_genes_per_cell=, min_cells =, num_cells=, batch_size=, partial_post_processor	Yes
	rapids_scanpy_funcs.py	preprocess_in_batches	*Cudf*	Preprocessing	markers, min_genes_per_cell=, max_genes_per_cell=, min_cells_per_gene=, target_sum=, n_top_genes=, max_cells=	Yes
	utils.py	tf_idf(filtered_cells) logtf_idf(filtered_cells, pseudocount = 10**5):	*numpy* *scipy*	TF-IDF normalization	pseudocount=	Yes
	utils.py	Frequency-based peak selection	*numpy*	Retains the top N most frequent peaks in the count matrix	filter_peaks (n_top_peaks):	Yes
	utils.py	PCA	*cuml*	Performs a batched PCA by training on the first `*train_ratio*` samples and transforming in `*n_batches*` number of batches	adata, n_components=, train_ratio=, n_batches=, gpu=	Yes
	scATACseq_Tutorial4.ipynb	UMAP	-	Dimensionality reduction technique	n_neighbors=, n_pcs=, method=, min_dist=, spread=	Yes
	rapids_scanpy_funcs.py	Graph clustering	*cuGraph*	Performs Leiden Clustering using cuGraph	flavor=, resolution=	Yes
	utils.py	overlap	-	Checks if a genomic interval ('fragment') overlaps a gene, or some number of bases upstream/downstream of that gene	gene, fragment, upstream = 10,000, downstream = 0	Yes
	rapids_scanpy_funcs.py	scale	*cupy* *numpy*	Scales matrix to unit variance and clips values	normalized, max_value=	Yes
	rapids_scanpy_funcs.py	_regress_out_chunk	*cupy*	Performs several local linear regressions, replacing the data in the original chunk w/ the regressed result	—	Yes
	rapids_scanpy_funcs.py	normalize_total	*cupy*	Normalizes rows in matrix so they sum to `*target_sum.*	csr_arr, target_sum	Yes
	rapids_scanpy_funcs.py	regress_out	*cupy*	Use linear regression to adjust for the effects of unwanted noise and variation	normalized, n_counts, percent_mito, verbose=	Yes
	rapids_scanpy_funcs.py	filter_cells	*cupy*	Filter cells that have genes greater than a max number of genes or less than a minimum number of genes	sparse_gpu_array, min_genes, max_genes, rows_per_batch=, barcodes=	Yes
	rapids_scanpy_funcs.py	filter_genes	*cudf*	Filters out genes that contain less than a specified number of cells	sparse_gpu_array, genes_idx, min_cells=	Yes
	rapids_scanpy_funcs.py	rank_genes_groups	*cudf* *cupy*	Rank genes for characterizing groups	groups=, reference=, n_genes=	Yes
	rapids_scanpy_funcs.py	_cellranger_hvg	*cudf*	Calculating highly variable genes Identifying genes to filter	mean, mean_sq, genes, n_cells, n_top_genes	Yes
	rapids_scanpy_funcs.py	highly_variable_genes	*cellranger* *cudf*	Identifies highly variable genes using the 'cellranger' method	sparse_gpu_array, genes, n_top_genes=	Yes

### Pooled-cell ATAC-seq workflow

**Figure 1:**
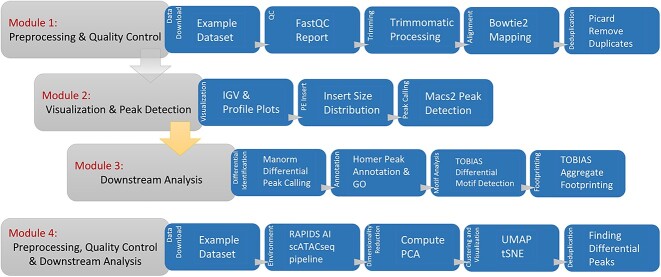
Describes the workflows adopted for pooled-cell ATAC-seq and sc-ATAC-seq analysis. Module 1 to Module 3 describes the preprocessing and quality control, visualization and peak detection, followed by downstream analysis for pooled-cell ATAC-seq workflow. Module 4 describes the preprocessing, quality control and downstream analysis for sc-ATAC-seq analysis.

The pooled-cell ATAC-seq workflow begins with *fastqc* [19] for performing quality control and *multiqc* [20] to summarize metrics. *Trimmomatic* [21] was used for trimming sequences, *Bowtie2* [22] for aligning reads and *Samtools* [23] for post-processing alignments. *Picard* [24] was used for manipulating formats and marking duplicates. *DeepTools* [25] was used to generate coverage files, calculate scores, and shift reads. *MACS2* was used to identify peaks, *Manorm* [26] for quantitating signal differences and *HOMER* [27] for annotating peaks. *TOBIAS* [28] was used for correcting bias, calculating footprint scores, and detecting motifs. Finally, *TOBIAS* [28] was also used for visualizing average footprints at TF motifs using *PlotAggregate* function. These tools played crucial roles in quality control, trimming, alignment, post-processing, peak identification, differential analysis, annotation and visualization throughout the ATAC-seq analysis framework (Figure 1).

### scATAC-seq workflow

The scATAC-seq analysis incorporates several custom Python files and functions. The *scATAC-seq_Tutorial4.ipynb* file defines variables and performs filtering on peaks, performs Principal Component Analysis (PCA), t-Distributed Stochastic Neighbor Embedding (t-SNE), K-Nearest Neighbors (KNN), Uniform Manifold Approximation and Projection (UMAP), differential peak analysis, and marker gene identification. The essential parameters used include “n_top_peaks”, “n_components”, “tsne_n_pcs”, “n_neighbors”, “knn_n_pcs,”, “umap_min_dist”, “umap_spread”, “n_diff_peaks” and “markers”. The *rapids_scanpy_funcs.py* custom Python file contains functions such as “read_with_filter” for reading h5ad files and applying cell and gene count filters, “preprocess_in_batches” for preprocessing with parameters like “markers” and various thresholds, “scale” for scaling matrices, and “regress_out” for adjusting for unwanted noise and variation. Parameters used include “min_genes_per_cell”, “max_genes_per_cell”, “min_cells”, “num_cells”, “batch_size”, “pseudocount”, “n_top_peaks,” and “target_sum”. The *utils.py* file includes the functions “tf_idf” and “logtf_idf” for term frequency-inverse document frequency (TF-IDF) normalization with parameters like “pseudocount”, and “filter_peaks” for frequency-based peak selection and “PCA” for batched PCA with parameters such as “n_components”, “train_ratio” and “n_batches”. Additionally, the “overlap” function in “utils.py” checks genomic intervals' overlap with genes using parameters like “gene”, “fragment”, “upstream” and “downstream.” These custom Python files work in tandem and are based on RAPIDS-AI framework for performing tasks such as data preprocessing, normalization, dimensionality reduction, filtering, regression and peak selection. The parameters used in each function enable customization and tuning to achieve optimal results in the analysis.

### Interactivity and enhanced learning

Throughout the module, users interact with quizzes and flashcards implemented by the jupyterquiz [29] and jupytercards packages. Data is browsable fully inside the modules using the integrated genome viewer (IGV) [30] embedded within the notebooks. Results are often produced as interactive html reports loaded inside the notebook.

## RESULTS

### CloudATAC platform integrates framework execution with augmented learning

**Figure 2:**
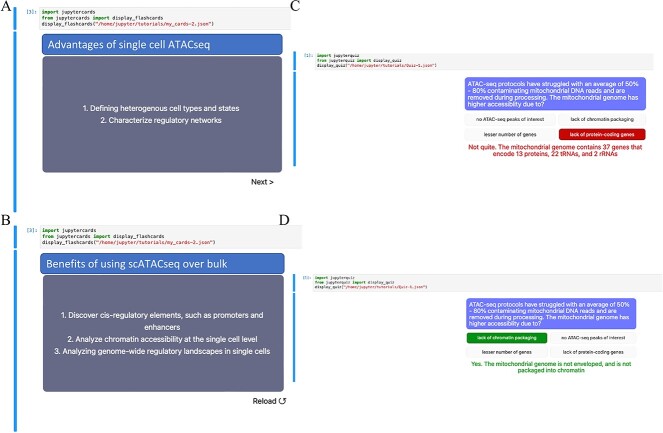
Shows the code snippets for displaying flashcards and quizzes to enhance learning while performing ATAC-seq workflows. **(A , B)** shows the code snippets of *jupyter cards* and its function *display_flashcards* used in displaying the front and back of flash cards. **(C, D)** shows the code snippets of *jupyter quiz* and its function *display_quiz* used in displaying the quiz.

Frameworks for pooled-cell and single-cell ATAC-seq data analysis were augmented using established and reliable programs [7] and divided into four modules (Figure 1) [17, 31]. Modules 1–3 host CPU-based workflows for pooled-cell ATAC-seq analysis, while module 4 hosts GPU-based workflows for single-cell ATAC-seq analysis. All steps in the workflow are implemented by executing live Python code blocks in Jupyter Notebook and subsequently visualizing the outcome. Every step within the modules is introduced with a clear explanation of its purpose. The outcomes of processing steps are then visualized in either a table, graph or image format. The modules enhance learning by interleaving workflow tasks with quizzes and flashcards, utilizing the *display_quiz* function from *jupyterquiz* for quizzes (Figure 2**A**) and the *display_flashcards* function from *jupytercards* for flashcards (Figure 2**B**). Each code block begins with a commented-out line that explains the code’s function, aiding users in understanding its utility. The Jupyter Notebooks for each module can be downloaded and customized to process local datasets. The source codes of these modules, along with example data, are publicly available through NIH Cloud lab https://github.com/NIGMS/ATAC-Seq-and-Single-Cell-ATAC-Seq-Analysis and are integrated with Google Cloud.

### Computational resources for ATAC-seq on the Google cloud

**Figure 3:**
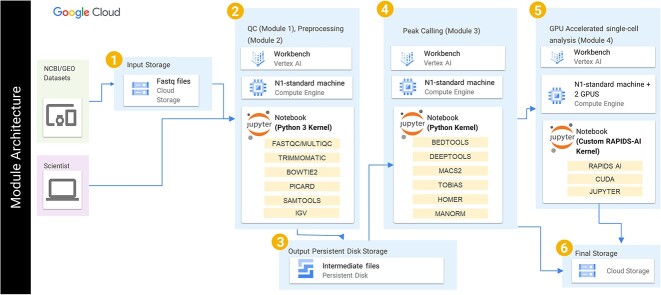
Shows the overall integrated roadmap that was used in augmenting both pooled-cell and sc-ATAC-seq workflows.

We used the Google Cloud to create a Vertex AI workbench. Modules 1 to 3 use *Python3* image on an *n1-standard-4* machine type having 4 vCPUs and 15 GB RAM. Additionally, we have created a custom image with Docker for module 4 with pre-requisite NVIDIA software and drivers installed for single-cell analysis. The *docker* image was created using Debian 10 OS architecture with an *n1-standard-8* machine type (8 vCPUs, 30 GB RAM) having *NIVIDIA T4* with 2 GPUs. The docker container with all dependencies, notebooks, and source code is available at

us-east4-docker.pkg.dev/nih-cl-shared-resources/nigms-sandbox/nvidiaforvertexai-rapids-22.12-cuda11.5-runtime-ubuntu20.04-py3.9@sha256:bb6703315633f21281e8caceed811f74822564a63ede01953664fe8d58b0c658Alternatively, *conda* can be used to create an environment and install dependencies. Since the scATAC-seq workflow is GPU-based, Unified Virtual Memory was configured to allow oversubscription of GPU memory, automatically offloading data chunks to main memory when needed, eliminating concerns about out-of-memory errors. The cloud services used in these modules are highlighted in the cloud architecture diagram shown in Figure 3.

### Optimization of ATAC-seq workflows

The ATAC-seq workflows are hosted within NIGMS Sandbox Github, alternatively it can also be run using Google Cloud implemented within NIH Cloud Lab. The workflows are invoked beginning with the setting up of the environment using the *conda* package manager to install requisite packages, copy example data files (raw ATAC-seq *fastq* and reference *fasta* files) from Google Cloud Storage Bucket (gs://nigms-sandbox/unmc_atac_data_examples/Tutorial1). Modules 1 to 3 utilize ATAC-seq data from a study by Bao, Rubin [17], which focused on human epidermal differentiation processes from adherent cells cultured *in vitro*. Sequences matching chromosome 4 were subsampled to facilitate learning and hasten the processing during the example tutorial framework. Module 1 contains a sequence of steps for performing quality check (QC), including fastqc and *multiqc*; preprocessing tasks involving trimming (*10eeptools10c*), mapping (*bowtie2*) and removal of PCR duplicates (*picard*); followed by interpretation of the results up to this stage.

**Figure 4:**
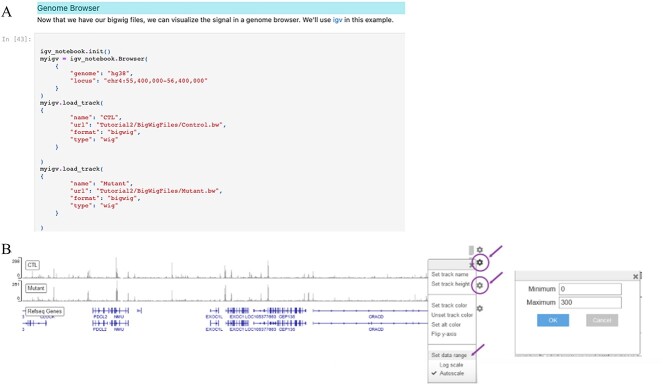
Displays the code snippet employed to enable the integrative genome browser and display the bigwig files to visualize ATAC-seq signals within the Jupyter Notebook.

In module 2, aligned reads undergo quality filtering using *samtools*. Bigwig format files summarizing the pileup of reads at each base pair are then created using *bamCoverage* from *the 10eeptools* package to visualize the results better. These bigwig files are loaded into a genome browser (IGV) embedded within the 10eeptool to visualize and interact with the ATACseq signals (Figure 4). Next, average profiles of ATACseq signals and distribution of insert sizes corresponding to accessible chromatin versus nucleosomal fragments are obtained using the *10eeptools* package. Peak detection is performed using *macs2,* providing a .narrowPeak file specifying the coordinates of the peaks, a .xls file with additional information, and a .bed file with the summits of the peaks.

**Figure 5:**
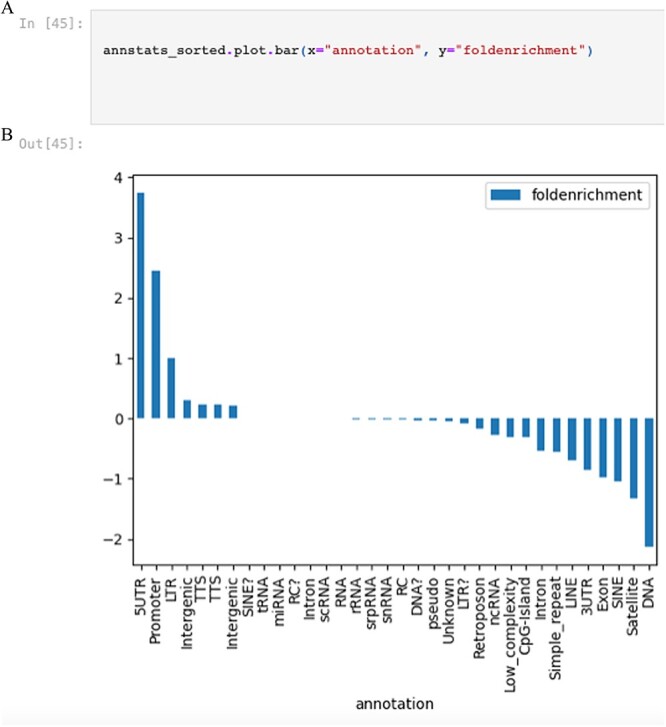
Displays the code snippet to illustrate the fold enrichment using a bar graph.

Module 3 focuses on downstream processing including differential peak identification, motif footprinting, and annotation of nearby genomic features. First, peaks and signal from the control and mutant are compared to identify a set of differential peaks using MANorm. Differential peaks are annotated to nearby genes and undergo gene ontology analysis (Homer) followed by visualization of term enrichments (Figure 5). Results within an HyperText Markup Language (HTML) file are loaded directly into the notebook to enable navigation through various ontology categories, allowing users to explore enriched terms and scores for genes near differential peaks. Lastly, this module introduces motif footprinting using *TOBIAS* on ATAC-seq data to identify accessibility at transcription factor (TF) binding sites. Tn5 insertion bias is corrected for the ATAC-seq data files, generating a bias-corrected bigwig file that is then used to calculate the footprint scores around the peaks. After calculating the footprint scores, TF binding site prediction and differential footprinting are performed using the reference motifs from *Jaspar*. The significance of the findings is visualized on an interactive volcano plot, which illustrates the differential binding score, differential TF and its corresponding motif sequence.

**Figure 6:**
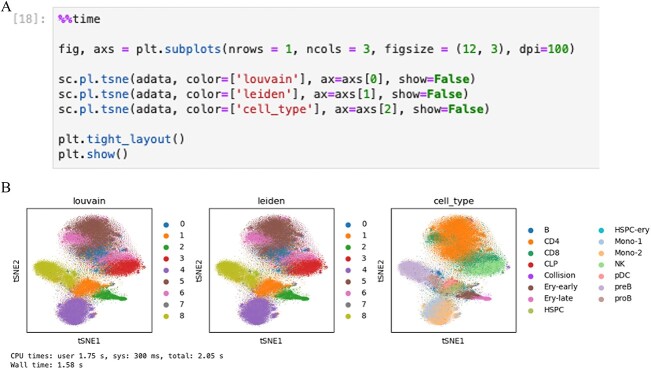
Shows the code using the *matplotlib* and *scanpy* libraries to create and display a figure with three subplots, each showing a t-SNE (t-Distributed Stochastic Neighbor Embedding) visualization with different colors representing different aspects of the data.

Module 4 demonstrates the utility of using *RAPIDS* for GPU-accelerated analysis of scATAC-seq data. We use single-cell ATAC-seq data from 60 495 cells that include data from the cells in the ‘Resting’ condition obtained from the study ‘Droplet Single-cell ATAC-seq of 60K Bone Marrow Cells’ by Lareau, Duarte [18]. In this module, each major step is accompanied by a code indicating the processing time for completion and overall framework progress. The framework starts with the peak-cell matrix and performs peak selection, normalization, dimensionality reduction, clustering and visualization. We also visualize regulatory activity at marker genes and compute differential peaks. *RAPIDS* framework uses NVIDIA *cuda*, *cudf* and *cuml* python libraries to improve performance, speed, fluid interaction with data, and cost. RAPIDS libraries begin from a single-cell count matrix to perform data processing, dimensionality reduction, clustering, visualization and comparison of cell clusters. With RAPIDS, it becomes easy to analyse large datasets interactively and in real-time, enabling faster scientific discoveries. The environment for module 4 is set up by installing *gcloud*, including *gsutil* and other Google command line tools. Data files such as processed peak-cell count matrix (.h5ad), set of nonzero peak names (.npy) and cell metadata (.csv) from the study by Lareau [32] were obtained from Google Cloud Storage bucket (gs://nigms-sandbox/unmc_atac_data_examples/Tutorial4). Module 4 begins by utilizing the Hierarchical Data Format version 5 (H5AD/HDF5) file that was originally derived from the Cell Ranger framework containing per-molecule information to support large, complex, and heterogeneous data. We have installed the custom Python functions developed by the NVIDIA research team in two files: ‘*rapids_scanpy_funcs*’ and ‘*utils,*’ followed by configuring the GPU memory to allow oversubscription. In the next step, we defined example parameters for filtering peaks, conducting PCA, t-SNE, KNN and UMAP, and identifying the number of differential peaks. Additionally, we designate marker genes along with their coordinates (Supplementary Figure 1). This follows the preprocessing steps such as TF-IDF normalization with which the PCA is performed, followed by frequency-based peak selection. In the subsequent step, clustering functions such as Louvain or Leiden are applied to group the cells, followed by visualization of the clustering results (Figure 6). Additionally, dimensionality reduction techniques (t-SNE) are employed to aid in visualizing high-dimensional datasets. The next step in the module computes the gene activity score representing the activity of each marker gene in each cell to find which peaks overlap with each marker gene (+ 5 kb upstream) using the *adata_raw* object. Furthermore, per-cell gene activity scores are calculated for each marker gene, and these scores are stored along with the annotated data (*adata*) for each cell and visualized using a UMAP plot indicating the activation of cell-type specific marker genes correlating well with the cell types identified by Louvain clustering. In the last step, we perform accelerated logistic regression-based differential peak computation using RAPIDS from *adata* generated in the previous step (Supplementary Figure 2). The statement *print(‘Full time: %.2fsec’ % (time.time() – start_time))* at the conclusion of module 4 will display the overall time elapsed from the initiation of the first code block until the end. In our evaluation using RAPIDS for single-cell ATAC-seq data analysis, the duration was approximately 23 minutes with an estimated cost of $3.80. This evaluation was conducted on a dataset comprising 60 495 cells. The analysis workflow included steps like peak selection, normalization, dimensionality reduction, clustering and visualization, extending to regulatory activity visualization at marker genes and computation of differential peaks. This contextualizes the cost and computational resources for a relatively complex and sizable dataset.

*Jupyter Notebooks* were chosen to develop the ATAC-seq analysis framework to enable iterating on algorithms and visualizations to deliver an end-to-end workflow. While we recognize workflow languages like CWL and Snakemake have advantages for portability and maintenance long-term, implementing the workflows in these would have lacked the interactive visualizations that are critical for non-bioinformaticians to understand and execute the framework. The interactive notebooks allowed us to optimize framework while benefiting non-technical users. Additionally, the combination of code, visuals, and narration in the *Jupyter notebooks* has intrinsic benefits for teaching the analysis concepts underlying the workflow.

### Integrating with the NIH cloud lab

Lastly, the ATAC-seq modules 1 to 4 were packaged and pushed into a GitHub repository hosted by NIGMS Sandbox alongside 11 additional biomedical workflows. A Google Cloud account is required to execute the modules. Accounts can be provisioned using Cloud Lab services or an individual domain. The sandbox-like environment offered by NIH Cloud Lab was utilized to leverage the Google Cloud’s capabilities for storage, execution, scalability, flexibility, and security. The NIH Cloud Lab GitHub repository contains additional Google Cloud tutorials.

### FAIR evaluation

To evaluate the equity and accessibility of the data and programs in the NIH Sandbox, we conducted a FAIR assessment. The FAIR principles outline best practices for scientific data management by promoting data that are Findable, Accessible, Interoperable and Reusable. The results of this FAIR evaluation revealed is given below.

### Findable

- Findable: CloudATAC is publicly discoverable and searchable online ensuring it is easily findable in GitHub.
- Metadata and Keywords: Comprehensive metadata and relevant keywords are included for easy discovery in search engines and databases.

Accessible

- Repository and Access: CloudATAC is hosted on a public repository of NIH STRIDES GitHub https://github.com/STRIDES with open access for users to download and utilize.
- Documentation: Detailed documentation is provided here https://github.com/NIGMS/NIGMS-Sandbox, outlining installation, usage, and troubleshooting, ensuring accessibility for a diverse user base.

Interoperable

- Data Formats: CloudATAC supports standard bioinformatics data formats, facilitating interoperability with other tools and databases.
- APIs and Integration: The framework incorporates APIs for integration with other bioinformatics tools and platforms, enhancing its interoperability within the scientific ecosystem.

Reusable

- License: CloudATAC is released under a permissive license under a Creative Commons CC-BY-NC-SA promoting reuse by the scientific community.
- Extensibility: The software architecture is designed to be modular, allowing for updates, expansions, and customization, fostering long-term reusability.

Analysis Reproducibility To enable reproducibility and transparency of the CloudATAC workflows, we have taken several steps aligned with FAIR principles. The *Jupyter Notebooks* containing the complete code, annotations, narration, and visualizations are version-controlled on *GitHub*. Users can download and execute the notebooks with sample data to replicate the analysis. We provide the Docker containers used for the runtime environments on *GitHub* as well. The notebooks document the key algorithms utilized through code, commentary, and citations. The workflows operate on public ATAC-seq datasets from Bao, Rubin [17] and Fang, Preissl [31]. Tables 2 and 3 outline the various tools and parameters involved in processing and analyzing ATAC-seq data for our best practices approach. Moving forward, we plan additional actions to further enhance the FAIRness and sharing of CloudATAC, including registering the workflows on repositories like Dockstore for improved discoverability and assigning digital object identifiers to workflows and containers in our next version release.

## DISCUSSION

ATAC-seq analysis, like other NGS technologies, demands significant computational and expert personnel resources. To harness the scalability and flexibility provided by the Google Cloud, we optimized the ATAC-seq and scATAC-seq workflows within a cloud environment and integrated it with an interactive front using the Jupyter Notebook. The workflows in these modules incorporate current "best practices" of using suitable tools with appropriate parameters and compatible resources alongside learning. These modules have been thoroughly tested and independently verified by teams at Google and Deloitte before being hosted by NIH Cloud lab. NIH Cloud Lab offers a sandbox-like environment for researchers to explore workflows in the cloud, strengthen training, prototype new architectures, and benchmark costs for research analyses.

We streamlined the pooled-cell ATAC-seq workflow by bringing the necessary entities together in one place. In this module, we capitalized on the versatility of employing multiple programming languages within the Jupyter Notebook. This platform seamlessly integrates with diverse tools and applications, providing open-source and cloud-based programming capabilities [13]. We successfully installed all the necessary packages and their dependencies, ensuring seamless integration from the initial step to the final one, including visualizing peaks on the genome browser. Regarding the scATAC-seq workflow, we focused on enhancing multiple aspects, including speed, storage, security, visualization, and learning of ATAC-seq data analysis. To address these areas, we incorporated RAPIDS-AI libraries [33] in the Google Cloud environment and accommodated them in the NIH Cloud lab. By harnessing the power of GPU acceleration from RAPIDS, we achieve accelerated data loading, preprocessing, feature engineering, modeling, and analysis for scATAseq data. The RAPIDS environment contains various libraries and frameworks, such as cuDF for data manipulation and processing, cuML for machine learning algorithms, cuGraph for graph analytics and cuSignal for signal processing. These libraries are designed to perform computations on GPUs, leveraging parallel processing and memory optimization to deliver faster and more efficient data processing compared to traditional CPU-based approaches [33]. We compared CloudATAC framework with some general-purpose bioinformatics workflow platforms like Galaxy, Terra and Nextflow Tower. Table 1 lists the distinct advantages of running CloudATAC in Google Cloud compared to general-purpose bioinformatics workflow platforms. Some key advantages of CloudATAC framework include preconfigured tools and workflows optimized for ATAC-seq data out-of-the-box, without needing to build workflows from scratch. Integration of interactive modules, quizzes and visualizations for enhanced ATAC-seq learning. Leveraging scalable cloud resources and GPU acceleration and tight integration with Jupyter Notebook for an interactive, reproducible environment.

In summary, we have augmented the pooled-cell ATAC-seq and sc-ATAC-seq frameworks using appropriate tools with best practices approach and deployed them on VertexAI virtual machines using RAPIDS-AI to accelerate the process. The frameworks are accessible through a Jupyter Notebook front-end [13], allowing easy code execution and visualization. It is hosted on the NIH Cloud Lab on the Google Cloud, providing a platform that eliminates the limitations of computing infrastructure, storage, network bandwidth, and the need for specialized IT and bioinformatics personnel. These frameworks facilitate seamless analysis of ATAC-seq data, enabling users to enhance their learning and test their knowledge conveniently and efficiently (Figure 3). The NIH cloud lab repository plays a crucial role in showcasing the modules for educating students and researchers on harnessing the potential of cloud technology to advance life sciences applications and research. This work utilizes the computational resources of the NIH STRIDES Initiative (https://cloud.nih.gov).

**Key Points**

- We present CloudATAC, an open-source cloud workflow for ATAC-seq data analysis leveraging the best practices approach, Google Cloud, Jupyter Notebook, and NIH Cloud Lab in one place.
- CloudATAC represents the first instance of streamlining the ATAC-seq workflows into a unified entity.
- We utilize RAPIDS-AI libraries to leverage GPU instances, significantly improving the run-time of the single-cell ATAC-seq workflow.

## Supplementary Material

Supplementary_Figure_1_bbae090

Supplementary_Figure_2_bbae090

## Data Availability

The source codes and data underlying this article are publicly available through the NIH Cloud lab at https://github.com/NIGMS/ATAC-Seq-and-Single-Cell-ATAC-Seq-Analysis

## Notes

**Avinash Veerappa** is an Instructor at the Genetics Cell Biology and Anatomy, University of Nebraska Medical Center. He currently works on developing new frameworks to model biological systems and to study the complexities of human diseases and disorders.

**M Jordan Rowley** is an Assistant Professor at the Genetics Cell Biology and Anatomy, University of Nebraska Medical Center. His research interests involve a broader field of epigenetics and chromatin biology, aiming to unravel the intricate mechanisms governing 3D chromatin organization and its impact on gene regulation and cellular processes.

**Angela Maggio** is a Biomedical Data Scientist & HealthTech Product Manager at Deloitte. Her work spans across data science, bioinformatics, technology strategy, and project management.

**Laura Beaudry** is a Program Manager at Google. Her work focuses on working with engineering teams to design and implement cloud solutions for federal agencies and organizations.

**Dale Hawkins** is a Technical Program Manager, Professional Services at Google Public Sector. His work involves coordinating new product launches, collaborating with cross-functional teams, and implementing efficient project management processes.

**Allen Kim** is a Cloud AI/ML Engineer at Google. He is an experienced tech professional specializing in Machine Learning and Data Science.

**Paul L Sorgen** is currently a Professor at the Department of Biochemistry and Molecular Biology, University of Nebraska Medical Center. His research interests involve understanding changes in gap junction channels, specifically Cx43 and Cx45, impact cardiac arrhythmias in heart diseases.

**Chittibabu Guda** is currently a Professor at the Genetics Cell Biology and Anatomy, University of Nebraska Medical Center. His research areas include developing novel algorithms for biomolecular data analysis, building data analysis frameworks for ‘omics' datasets, and conducting integrative analysis of cancer genomics data.
