# Supplementary figures and images for: CloudATAC: a cloud-based framework for ATAC-Seq data analysis
Source: Brief Bioinform. 2024 Jul 23;25(Suppl 1):bbae090. doi: 10.1093/bib/bbae090 (PMC11264300; doi:10.1093/bib/bbae090)

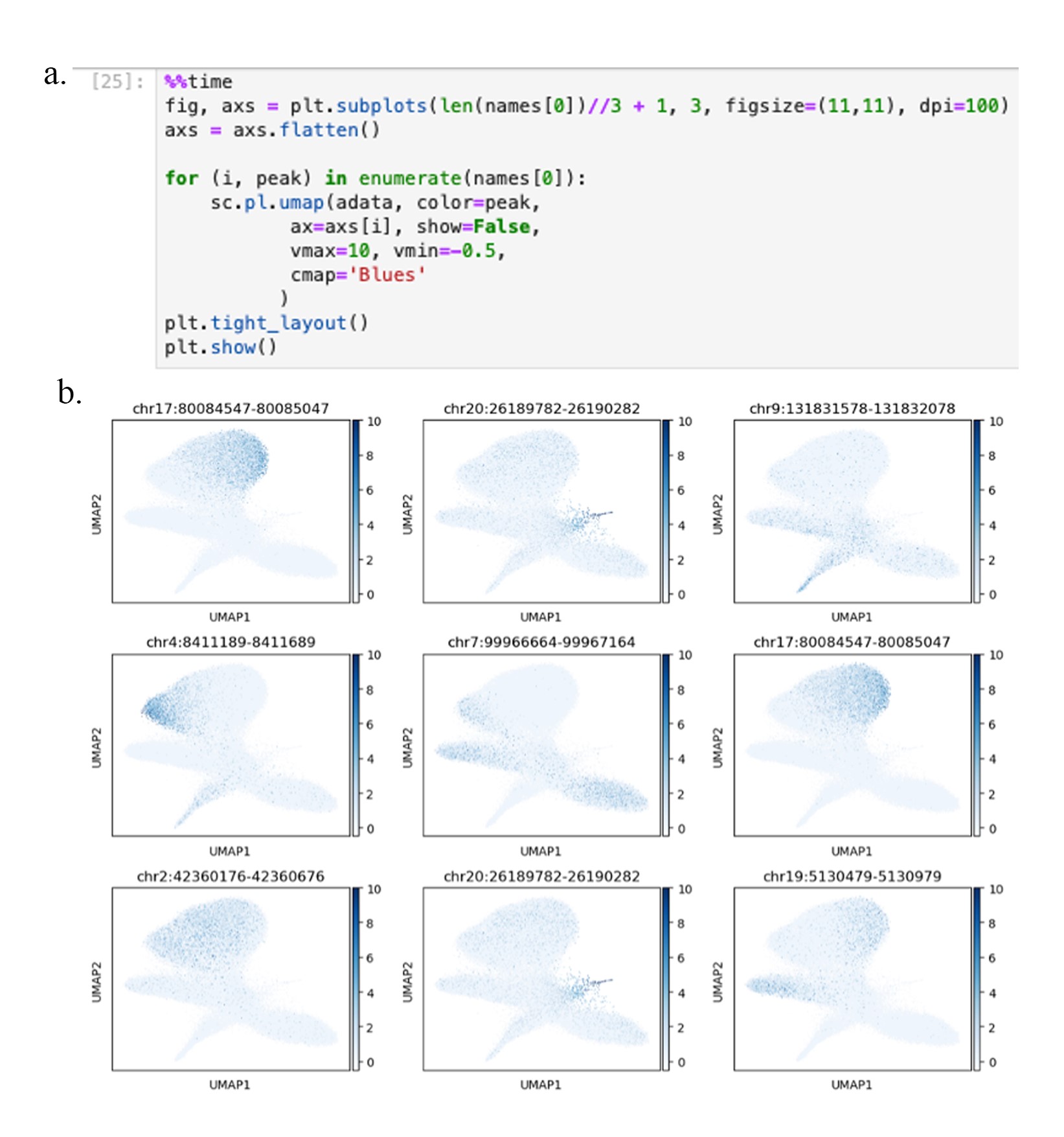

Supplement: Supplementary_Figure_1_bbae090 [file supplementary_figure_1_bbae090.jpeg]

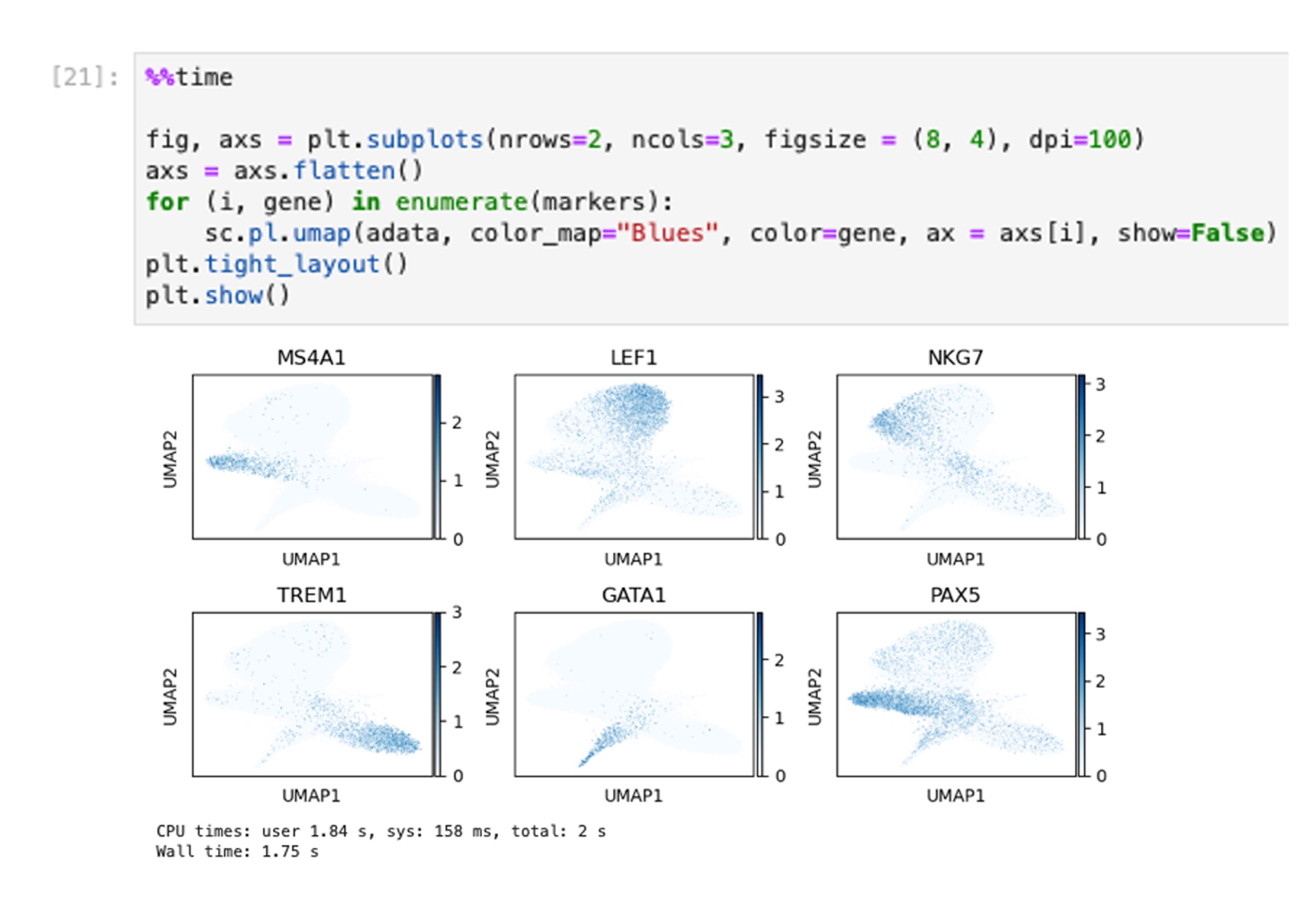

Supplement: Supplementary_Figure_2_bbae090 [file supplementary_figure_2_bbae090.jpeg]
